# An Approach to Catheter Ablation of Cavotricuspid Isthmus Dependent Atrial Flutter

**Published:** 2006-04-01

**Authors:** Mark D O’Neill, Pierre Jais, Anders Jönsson, Yoshihide Takahashi, Frédéric Sacher, Mélèze Hocini, Prashanthan Sanders, Thomas Rostock, Martin Rotter, Jacques Clémenty, Michel Haïssaguerre

**Affiliations:** Hôpital Cardiologique du Haut Lévêque & Université Victor Segalen Bordeaux II, 33604 Bordeaux, Pessac, France.

**Keywords:** Atrial flutter, cavotricuspid isthmus, ablation

## Abstract

Much of our understanding of the mechanisms of macro re-entrant atrial tachycardia comes from study of cavotricuspid isthmus (CTI) dependent atrial flutter. In the majority of cases, the diagnosis can be made from simple analysis of the surface ECG. Endocardial mapping during tachycardia allows confirmation of the macro re-entrant circuit within the right atrium while, at the same time, permitting curative catheter ablation targeting the critical isthmus of tissue located between the tricuspid annulus and the inferior vena cava. The procedure is short, safe and by demonstration of an electrophysiological endpoint - bidirectional conduction block across the CTI - is associated with an excellent outcome following ablation. It is now fair to say that catheter ablation should be considered as a first line therapy for patients with documented CTI-dependent atrial flutter.

## Introduction

Since the first description of atrial flutter in humans almost a century ago, electrophysiological techniques have evolved to facilitate a comprehensive understanding of the arrhythmia mechanism which in turn, has led to a highly effective therapeutic strategy. This review is confined to cavotricuspid isthmus-dependent flutter and details the development of the understanding, diagnosis and therapy of this common arrhythmia. Specifically, the approach used for the catheter ablation of atrial flutter at our institution is described in detail.

## Insights from electrocardiography

Early recognition of the difference between atrial flutter (AFL) and atrial fibrillation (AF) was made by Jolly and Ritchie with the demonstration of regular atrial activity in the inferior leads of the surface electrocardiogram. Lewis and colleagues demonstrated in animal studies that constant and ordered activation of at least part of the atrium was responsible for the flutter waves on the surface ECG and remarkably deduced that atrial flutter was likely due to intra-atrial circus movement around the venae cavae (for historical review, see Lee [1]).

Cavotricuspid isthmus-dependent counterclockwise flutter is the archetypal atrial macro-re-entrant arrhythmia, the anatomical circuit of which has now been well described with both conventional and newer mapping modalities [2-5]. The route of the re-entrant circuit is bordered anteriorly by the tricuspid annulus and posteriorly by the venae cavae orifices, the crista terminalis and the Eustachian ridge [6]. The so-called cavotricuspid isthmus (CTI) serves as the critical zone of slowed conduction which facilitates perpetuation of the re-entrant circuit and also serves as an ideal target for its physical interruption by radiofrequency ablation [4-7].

**Figure 1:**
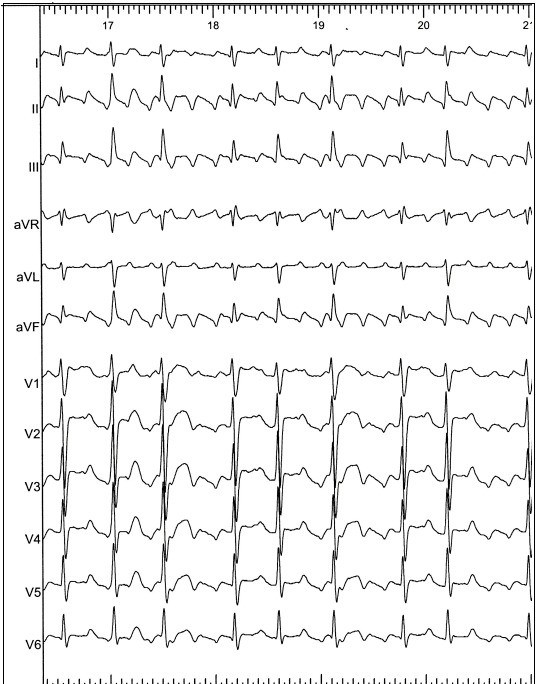
Twelve lead electrocardiogram of counterclockwise cavotricuspid isthmus dependent atrial flutter with a variable ventricular response rate. Note the presence of the negative “saw tooth” flutter waves in leads II, III and aVF in addition to the positive flutter wave in V1 with a transition of morphology across the anterior precordial leads.

Flutter waves are atrial complexes of constant morphology, polarity and cycle length in a rate range from 240 to 340 per minute [6] (Figure 1). The presence of flutter waves in the inferior limb leads and in V1 is specific for cavotricuspid isthmus-dependent flutter [6]: negative flutter waves in the inferior leads and a positive wave in V1 with transition of morphology across the anterior leads is consistent with a counterclockwise atrial circuit with lateral-to-medial activation of the cavotricuspid isthmus (CTI) while activation in the reverse direction (clockwise) is suggested by positive flutter waves in the inferior leads and a negative deflection in V1, which accounts for 5-10% of all CTI-dependent flutter. There are rare forms of tachycardia with similar P wave morphology, for example atrial tachycardia originating from the ostium of the coronary sinus. Endocardial mapping in this case will demonstrate caudo-cranial activation of both the RA septum and lateral wall, which is not in keeping with counter-clockwise rotation around the previously described flutter circuit.

An important subgroup of patients to consider are those presenting with atrial flutter following catheter ablation of the left atrium for treatment of atrial fibrillation. It has recently been shown that over 50% of patients with electrophysiologically confirmed counterclockwise CTI-dependent flutter as the arrhythmia of recurrence following LA ablation for AF show upright flutter waves in the inferior leads, a finding which is most likely due to the left atrial debulking and resultant voltage abatement among this group [8]. Therefore, while the presence of negative flutter waves in the inferior leads is highly suggestive of CTI dependent flutter, their absence does not exclude the diagnosis, most notably in the context of structural heart disease or prior left atrial ablation.

## Electrophysiological evaluation of CTI-dependent flutter

### Coronary sinus activation sequence

**Figure 2:**
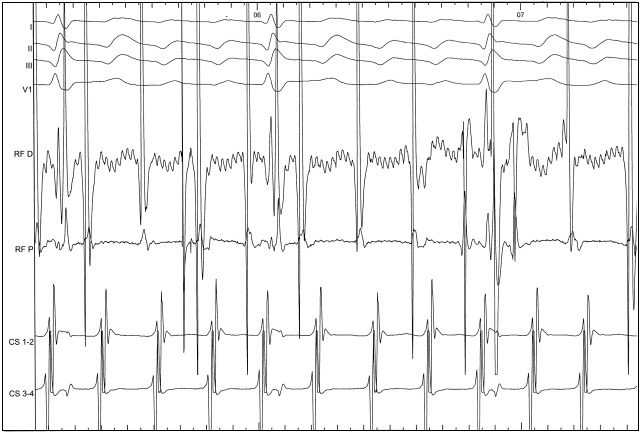
The upper aspect of the figure shows leads I, II, III and V1 of the 12 lead electrocardiogram. The middle two tracings are the endocardial signals recorded at the distal (RF D) and proximal (RF P) bipoles of the ablation catheter. The lower pair of tracings shows the bipolar electrograms recorded via a quadripolar catheter positioned with CS 3-4 at the ostium of the coronary sinus (CS). Note the coincidence of the atrial electrogram on RF D with the centre of the plateau of the flutter wave, indicating a medial position of the ablation catheter on the cavotricuspid isthmus (CTI). The activation sequence in the CS is from proximal to distal as expected for counterclockwise CTI-dependent flutter.

Following a thorough evaluation of the surface ECG, a quadripolar deflectable diagnostic catheter (Xtrem, ELA Medical) and mapping/ablation catheter are advanced from the right femoral vein to the coronary sinus (CS) and right atrium respectively. The distal pole of the CS catheter is positioned at 4 to 6 o’clock in the LAO position. If the patient is in AFL at the time of the study, the coronary sinus activation sequence should be from proximal to distal (Figure 2), although equally this could be the case for any tachycardia mechanism originating in the right atrium, and may also be seen in some left atrial flutters, for example counterclockwise perimitral flutter. Conversely, flutter localized to the superior right atrium may activate the distal CS earlier than the proximal CS, via Bachmann’s bundle. Together with the typical ECG findings, a proximal-to-distal CS activation sequence is not diagnostic for, but rather is highly suggestive of CTI-dependent flutter as the underlying tachycardia mechanism.

### Endocardial mapping

Once the diagnosis of CTI-dependent flutter is suspected from the surface ECG and the CS activation sequence, more detailed intra-atrial mapping is performed. Using the CS as a stable reference, up to eight points are sequentially mapped within the right atrium, including approximately four points around the tricuspid annulus, and their timing relative to flutter wave onset and the clearest CS signal determined, to evaluate the direction of impulse propagation during tachycardia. For the majority of patients with CTI-dependent counterclockwise flutter, the atrial electrogram recorded on the mid-isthmus lies within the plateau phase of the flutter wave, between 50 and 90ms prior to the onset of the pronounced negative deflection associated with the septal ascent of the depolarisation wavefront. Demonstration of a right atrial activation time which is significantly shorter than the tachycardia cycle length or the identification of colliding activation fronts in the right atrium are features which are highly suggestive of a left atrial flutter origin [5].

### Entrainment manoeuvres and post pacing interval

In addition to this activation mapping, entrainment manoeuvres can be employed in the right atrium and coronary sinus, to confirm and exclude the right and left atria as the chamber of arrhythmia origin respectively. Entrainment involves pacing from multiple, separate sites within the right atrium at cycle lengths of 10-20ms faster than the tachycardia cycle length, observing its effect on flutter wave morphology [9] and estimating proximity of pacing site to tachycardia circuit by analysis of the post pacing interval. The pacing site is considered to lie within the tachycardia circuit when the post pacing interval is within 30ms of the tachycardia cycle length. Entrainment from sites which are outside the flutter circuit will demonstrate manifest fusion on the surface ECG and the PPI will exceed the flutter cycle length by more than 30ms. Pacing performed from within the circuit but outside a protected isthmus demonstrates manifest entrainment, characterized by surface ECG fusion, progressive fusion at faster pacing rates and all sites which are orthodromically activated having a PPI equal to the flutter cycle length. Transient entrainment performed from within the protected CTI isthmus demonstrates a long stimulus-to-p wave interval, in keeping with its role as an area of slow conduction in which unidirectional block of the antidromic wave front probably occurs. Mapping of the septal aspect of the CTI demonstrates the earliest atrial electrogram relative to the onset of the flutter wave on the surface ECG, and entrainment from this position demonstrates a short stimulus-to-p wave interval with concealed fusion, confirming this as the likely exit site from the zone of slow conduction critical to maintenance of the tachycardia. This manoeuvre is repeated for a minimum of two right atrial sites, usually incorporating the RA septum and lateral wall at the very least.

Many patients with a history of atrial flutter who been referred for ablation therapy are in sinus rhythm at the time of their procedure. If the 12 lead electrocardiogram recorded during the clinical arrhythmia is consistent with CTI-dependent flutter, and the medical history gives no cause to suspect a left atrial origin, it is our practice to proceed directly to radiofrequency ablation of the CTI rather than to attempt induction.

## Catheter ablation of CTI-dependent flutter

Electrophysiological study and ablation is performed in the post absorptive state and under light sedation (midazolam 2-5mg and/or analgesia (nalbuphine 10 to 20mg). All antiarrhythmic medication, with the exception of amiodarone, is discontinued at least five half lives prior to the procedure. For patients already receiving oral anticoagulation, this is discontinued 48 hours prior to the procedure and continued for one month after the procedure. Otherwise, patients do not routinely receive anticoagulation medication before, during or after an elective CTI ablation.

All surface ECG leads are recorded continuously throughout the procedure to facilitate rapid review however only leads I, II, III and V1 are displayed in real time. Bipolar intracardiac electrograms are recorded in the coronary sinus using a quadripolar deflectable catheter and from the atrial endocardium using a conventional quadripolar thermocouple D curve 8mm tip catheter (Biosense Webster). All electrograms are sampled at 1KHz, filtered from 30 to 500Hz, amplified at 0.1mV/cm and displayed at a sweep speed of 100ms using a Windows-based digital amplifier/recording system for offline analysis (Bard Electrophysiology).

Ablation of the CTI is performed in the patient’s presenting rhythm, be it flutter or sinus rhythm. In the case of the latter, pacing is performed from the proximal pole of the CS (cycle length 600ms) as this location is more stable than a pacing catheter positioned in the inferolateral right atrium.

### The anatomical target

It is now well established that the anatomical target for interruption of the macro re-entrant circuit comprising typical atrial flutter is the cavo-tricuspid isthmus [4,5]. Using an 8mm tip ablation catheter, the linear lesion is made by a continuous application of RF energy beginning at the ventricular end of the CTI (A:V electrogram amplitude ratio of ~1:2) and dragging the catheter towards the atrial aspect of the CTI with a 60-90s delay at successive ablation points before moving the catheter. Radiofrequency energy (550Hz unmodulated sine-wave output) is delivered through a Cordis Stockert generator in temperature control mode with the temperature limited to 60°C and the power to 60W. Ablation is not routinely performed at the septal aspect of the CTI or inside the ostium of the CS in order to minimize the risk of circumflex artery injury, atrioventricular block or cardiac perforation. Nevertheless, lateral or septal positions are occasionally used when linear block cannot be achieved by ablation in the medial isthmus.

At our institution, an irrigated 3.5 mm tip catheter is used in all patients undergoing pulmonary vein isolation and left atrial ablation for atrial fibrillation. Unless there is evidence of persisting bidirectional conduction block across the CTI from a previous catheter intervention, it is our practice to ablate the CTI routinely in these patients following completion of the left atrial procedure. In those patients admitted electively for conventional CTI ablation for typical flutter, a non-irrigated 8mm tip catheter is used (Cordis Webster D curve).

The length, depth and anatomical complexity of the cavotricuspid isthmus can vary considerably between patients with a resultant impact on the success of radiofrequency ablation [10,11]. To facilitate effective ablation at the ventricular aspect of a “long” CTI, a long sheath can be used. Similarly, where there is difficulty in completing the IVC end of the line because of a prominent Eustachian ridge preventing a smooth drag back across the isthmus, the catheter may be looped and apposed to the isthmus from the atrial side and advanced from the IVC towards the Eustachian ridge. In patients where conduction block across the isthmus cannot be achieved with a conventional catheter, the use of an irrigated tip catheter has been shown to facilitate achievement of bidirectional CTI conduction block [12]. Interestingly, there is a significant correlation between the surface atrial ECG amplitude and the amount of RF energy required to achieve complete isthmus block, a finding which may be of use in the preprocedural choice of ablation catheter for typical atrial flutter [13]. Paradoxically, higher voltages recorded at the CTI using an electroanatomic approach do not correlate with reduced ability to achieve bidirectional conduction block when ablation is performed with a conventional 8mm tip RF catheter [14].

### The electrophysiological target

**Figure 3:**
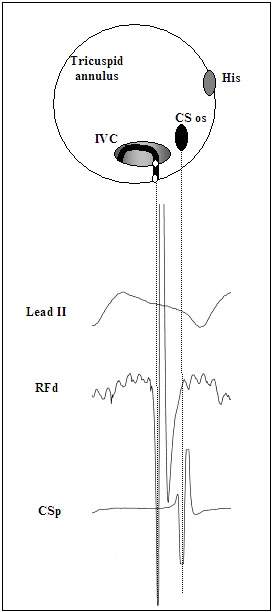
Cartoon depiction of the anatomical-to-electrophysiological relationship at the cavotricuspid isthmus (CTI) during CTI-dependent counterclockwise flutter. Shown are Lead II of the surface ECG, bipolar electrograms recorded at the distal pole of the ablation catheter (RFd) and the proximal pole of the coronary sinus catheter (CSp) positioned in close proximity to the CS os. Note the mid-plateau position of the ablation signal and the end-plateau position of the CSp signal, corresponding to the anatomical position of the mid and septal aspects of the CTI respectively.

The flutter wave morphology in counterclockwise CTI dependent flutter is characteristic, consisting of a downsloping “plateau” phase, followed by a short, sharp negative deflection, then a sharp positive deflection with a positive ‡overshoot’ which in turn leads to the next plateau. The anatomical location of the medial part of the CTI has been shown to correlate well with the timing of the atrial electrogram relative to the plateau of the flutter wave and ablation targets the atrial electrogram occurring simultaneously with the middle of the plateau phase of the flutter wave in lead II (Figure 2 & 3). When ablation is performed during flutter, careful attention to this relationship ensures radiofrequency energy is delivered perpendicular to the advancing wavefront and allows immediate recognition of displacement of the catheter medially or laterally, based on delay or advancement of the atrial electrogram respectively, relative to the plateau [15]. When performed during proximal coronary sinus pacing, RF energy is sequentially delivered at electrogram sites in the isthmus region with a constant stimulus-to-atrial electrogram time across the length of the isthmus.

### The end point

**Figure 4:**
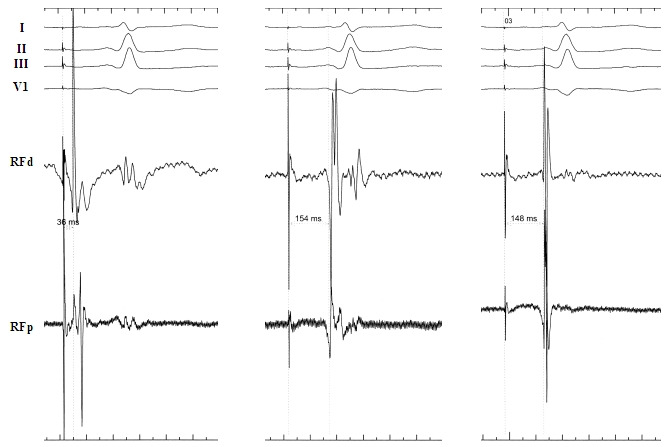
Verification of conduction block in a clockwise direction across the CTI. Pacing is performed at the proximal coronary sinus. In the left panel, the mapping/ablation catheter (RF) is placed medial to the line of block confirming a short delay (36ms) from the pacing site. In the middle panel, the RF catheter is placed on the ablated CTI, documenting a delay from the pacing site of 154ms. By moving the RF catheter immediately lateral to the line of block, the delay shortens to 148ms, consistent with cranio-caudal activation of the lateral right atrium and at least unidirectional block of the CTI. Note also the reverse polarity of the electrograms recorded at RFd when moving from the CTI to a more lateral position.

**Figure 5:**
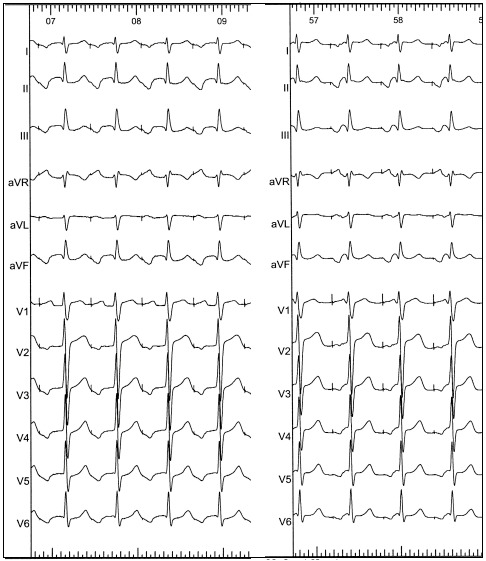
The left panel shows a 12 lead electrocardiogram following termination of the atrial flutter described in figures 1 and 2 but prior to achievement of bidirectional conduction block. The right panel shows a 12 lead electrocardiogram following completion of bidirectional conduction block. The stimulus artifact represents proximal coronary sinus pacing in both cases. The important feature to note is the increased positivity of the terminal component of the P wave in lead II in the right panel relative to the left. This represents cranio-caudal activation of the lateral right atrium which is now activated late relative to the septal RA and LA following achievement of conduction block across the isthmus.

Completion of the linear lesion by radiofrequency energy requires subsequent quantitative demonstration of bidirectional conduction block across the CTI in order to achieve the best outcomes for catheter ablation of atrial flutter. Complete bidirectional conduction block across the CTI may be defined as: a descending wave front on the lateral atrial wall up to the line of block during proximal CS pacing representative of a reversed atrial depolarization sequence; a greater activation delay measured to the second double potential recorded on the line of block when compared with the delay measured to the atrial electrogram further lateral to the line during proximal CS pacing (Figure 4); a decreasing activation delay measured medial to the line when pacing from immediately lateral to the line and from further lateral to the line [16]. Other electrophysiological features consistent with conduction block across the CTI include a reversal in bipolar electrogram polarity immediately lateral to the line during proximal CS pacing, signifying reversal of the vector of activation [17] (Figure 4); change in morphology of the unipolar electrogram (from QS, rS or RS to R or Rs) recorded immediately lateral to the line of block during proximal CS pacing [18]; mapping of a corridor of double potentials with an intervening isoelectric interval along the line of ablation [19,20]; demonstration of a reversal in the direction of atrial depolarization during proximal CS pacing either by sequential right atrial mapping with the ablation catheter or the use of a multipolar diagnostic catheter positioned along the lateral aspect of the tricuspid annulus. In practice, a craniocaudal activation of the septum (PCS activated after His bundle region) during pacing of the low lateral RA indicates an counterclockwise block in the isthmus, whereas a craniocaudal activation (low after high RA) of the lateral RA during pacing of the PCS indicates a clockwise block, and is reflected by increased positivity of the terminal component of the P wave in lead II (Figure 5). When taken together with an abrupt increase in the degree of separation of atrial bipolar electrograms recorded on the line of block, and the highly unlikely situation of ablation resulting in unidirectional clockwise conduction block [21], it is usually sufficient to document the atrial electrogram separation and confirm maximum conduction delay immediately medial to the line during low lateral right atrial pacing.

## Complications

Although uncommon, complications have been reported for ablation confined to the CTI. Injury to the right coronary artery has been described [22]. The atrioventricular (AV) node may be adversely affected by modification of local vagal afferent input related to RF stimulation or by a global vagal response to pain during energy delivery [23]. Of course, direct thermal injury to the node or its critical inputs may also result in transient or even permanent AV block.

## Conclusion

Radiofrequency ablation has been shown to be more effective than anti-arrhythmic medication in preventing recurrences of and reducing hospitalizations for CTI-dependent atrial flutter [24]. Catheter ablation is safe, cost-effective and should be considered as the first line treatment of choice for the majority of patients [25].
